# Multiplanar MRI approach to the differential diagnosis of central nervous system tumors and tumor-like lesions in patients with HIV: what radiologists need to know

**DOI:** 10.3389/fmed.2026.1779221

**Published:** 2026-03-20

**Authors:** Rustam Talybov, Tatiana Trofimova, Vladislav Spasennikov, Tatyana Kleschevnikova, Dzhamilia Murzaeva, Svetlana Kondratova, Yauhen Statsenko

**Affiliations:** 1 Tyumen State Medical University of the Ministry of Healthcare of Russia, Tyumen, Russia; 2 Regional Clinical Hospital No. 2, Tyumen, Russia; 3 First Pavlov State Medical University of St. Petersburg, St. Petersburg, Russia; 4 Department of Radiology, College of Medicine and Health Sciences, United Arab Emirates University, Al Ain, United Arab Emirates

**Keywords:** CNS, HIV infection, multiparametric MRI protocol, tumor-like lesions, tumors

## Abstract

HIV infection involves a wide range of diseases that affect the central nervous system (CNS). This variability makes diagnosing cerebral diseases difficult, especially when a tumor is unrelated to HIV. This review analyzes verified cases of CNS tumors and tumor-like lesions in HIV-positive patients. It focuses on original neuroimaging features for differential diagnosis. Given that cerebral pathology of any etiology frequently manifests as depressed consciousness and sometimes progressses to coma, the application of multiparametric magnetic resonance imaging (mpMRI) is essential for accurate diagnosis when the patient’s clinical history or physical examination results are limited. Herein, we discuss the specific neuroimaging patterns that have significant practical value for clinicians and radiologists. A correct diagnosis of a brain lesion is necessary to select proper treatment strategies, thus reducing mortality.

## Introduction

The World Health Organization (WHO) reports that 44.1 million people currently live with HIV in 2025. HIV infection is a systemic disease that can include HIV-related cerebral disease lesions (1, 2). The CNS becomes involved because of progressive immune function failure in late stages. This complication causes death in 40–70% of cases (3–6).

Diagnosing cerebral complications is often difficult. Patients may conceal their infection status. Changes on neuroimaging can also be nonspecific and variable (7, 8). In HIV-infected patients with acute neurological deficits and focal brain lesions, clinicians must differentiate between several conditions. These include cerebrovascular lesions, opportunistic neuroinfections, demyelinating diseases, and primary central nervous system large B-cell lymphoma (PCNS-LBCL) (7, 9–12). Radiologists may suspect a tumor or tumor-like lesion on an imaging study if they see specific features. These features include a solitary brain tissue lesion, perifocal vasogenic edema, a mass-effect, and contrast enhancement (9, 13, 14).

This article discusses clinical examples that produce comparable imaging findings. Noninvasive diagnosis poses a difficult task for identifying etiology (15, 16). A clinical misdiagnosis can lead to improper treatment and may delay a necessary biopsy (17, 18). MRI is a powerful noninvasive tool for identifying imaging patterns that may indicate specific etiologies of brain lesions. When combined with a patient’s clinical history and physical examination, imaging techniques can confirm or refute a preliminary diagnosis. This narrative review summarizes the most common imaging patterns of brain lesions in HIV-positive patients, as well as their distinctive neuroimaging and histological features. mpMRI of each lesion is also discussed.

### Laboratory findings of HIV-associated CNS diseases

The CD4-positive T-lymphocyte count is the most important prognostic marker. It assesses the risk of a cerebral lesion from HIV-infection progression. Patients with CD4 counts less than 200 cells/μL have a markedly elevated risk of demyelination, opportunistic infections, and PCNS-LBCL (7). For diagnostic verification, official guidelines recommend serological tests on blood serum and cerebrospinal fluid. They also suggest a polymerase chain reaction (PCR) test of the cerebrospinal fluid. In some cases, stereotactic biopsy is necessary to confirm the final diagnosis.

### Multiparametric MRI technique

Clinicians should use a mpMRI protocol for the differential diagnosis of patients with focal brain lesions (19). This approach integrates multiple pulse sequences, with each one providing distinct yet complementary data.

The standard mpMRI protocol for brain imaging includes several essential sequences. These include routine T1-weighted imaging (T1WI), T2-weighted imaging (T2WI), and T2-FLAIR. It also includes specialized sequences such as diffusion-weighted imaging (DWI) with apparent diffusion coefficient (ADC) maps, susceptibility-weighted imaging (e.g., SWI, SWAN, T2*), and dynamic susceptibility contrast (DSC) T2* -weighted perfusion.

The maximal slice thickness should be at least 5 mm in routine sequences, and 6 mm in T2* DSC. In certain cases, proton magnetic resonance spectroscopy (1H-MRS) may also be utilized (19–21). Each sequence acts as an independent diagnostic unit that together forms a comprehensive framework for analysis. This integrated protocol is therefore critical for accurate radiological assessment.

A fundamental understanding of tissue signal characteristics within each sequence is essential for accurate MRI interpretation. Radiologists must never rely on findings from a single sequence in isolation. Instead, they must correlate results across all sequences to enhance diagnostic precision. For example, a finding on DWI must be checked against T1, T2, and contrast-enhanced images. This cross-referencing is the core principle of the mpMRI methodology. Adhering to this practice ensures a robust and reliable diagnostic process.

The protocol also requires specific considerations for its advanced perfusion techniques. Motion artifacts, susceptibility artifacts, or contraindications to gadolinium-based contrast agents can sometimes limit DSC-T2* perfusion. In these scenarios, supplementary techniques such as arterial spin labeling (ASL) perfusion or CT perfusion provide valuable alternatives. Furthermore, precontrast administration of a small gadolinium dose is recommended before the main DSC perfusion scan. This technique saturates the blood–brain barrier (BBB) to minimize errors from contrast agent leakage in areas of BBB disruption (22–24). These refinements are crucial for acquiring high-quality, interpretable perfusion data.

Consequently, the meticulous application of the mpMRI protocol provides a powerful foundation for diagnosing cerebral lesions. The following discussion will detail the specific imaging patterns these sequences reveal.

### Tumor-like lesions

This section details common tumor-like lesions in HIV-positive patients, focusing on their etiology and key imaging features for differential diagnosis. Immunodeficiency from low CD4^+^ counts creates a high risk for opportunistic infections that can mimic tumors. We discuss the specific MRI characteristics of neurotoxoplasmosis, aspergillosis, cryptococcosis, and CNS tuberculosis. Correctly identifying these infections is critical to avoid unnecessary surgical intervention and to initiate appropriate antimicrobial therapy.

#### Neurotoxoplasmosis

**Figure 1:**
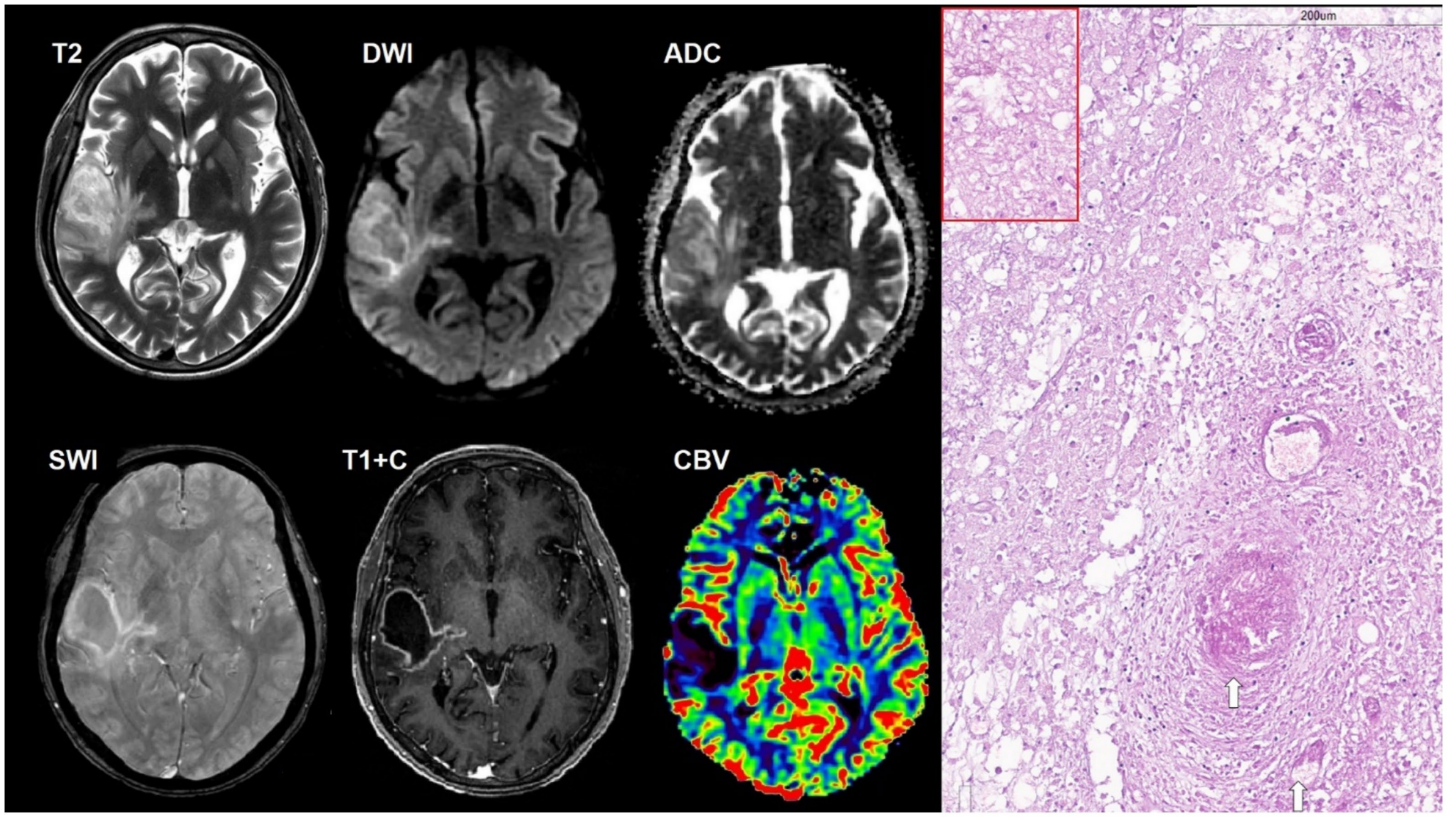
A 34-year-old HIV-positive male, with solitary cerebral toxoplasmosis, presented with a 4 day history of headache and left-sided weakness without an altered state of consciousness. The CD4^+^ lymphocyte count was 100 cells/μL. The titer of antibodies to *T. gondii* was 1:1,600. There was an intraaxial lesion in the right temporal lobe that a spread to the posterior parts of the subinsular region with ring-shaped contrast enhancement, mild restricted diffusion in the lesion walls and moderate perifocal edema. SWI does not indicate susceptibility artifacts. DSC-perfusion demonstrates low CBV values in the lesion. Microscopic characteristics: H&E staining; scale bar: 200 μm, applies also for inset in the red rectangle. Fibrinoid vascular necrosis (white arrows) and large foci of coagulation necrosis of the surrounding brain parenchyma with limited lymphocytic inflammation and macrophages. Red rectangle: free small basophilic tachyzoites of *Toxoplasma gondii* at the periphery of necrotic areas. Polymerase chain reaction (PCR) for *Toxoplasma gondii* was positive.

**Figure 2:**
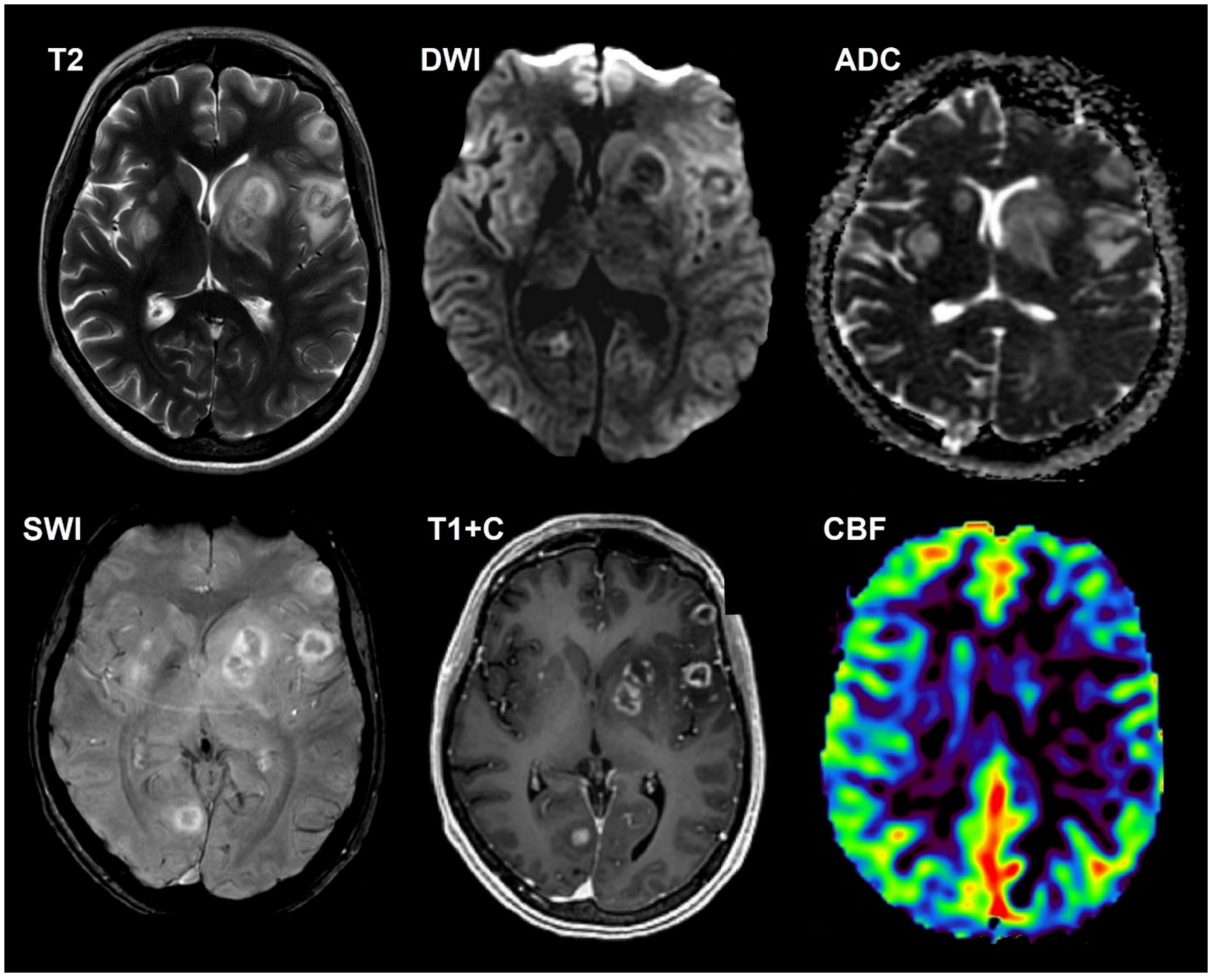
A 39-year-old HIV-positive woman, with multiple cerebral toxoplasmosis, a 4-week history of seizures and headaches was included. The CD4^+^ lymphocyte count was 200 cells/μL. The titer of antibodies to *T. gondii* was 1:400. There is multiple intracerebral lesion in with ring-shaped contrast enhancement (target-sign), increased diffusion restriction in the walls and mild perifocal edema. SWI does not indicate susceptibility artifacts. DSC-perfusion demonstrates low CBV values in the lesions. Polymerase chain reaction (PCR) for *Toxoplasma gondii* was positive.

Neurotoxoplasmosis is the most common opportunistic neuroinfection in immunocompromised patients, and is caused by the protozoan *T. gondii* (7, 25). It leads to cerebral abscesses that present with nonspecific clinical symptoms and a sluggish course (7, 25). In its acute necrotizing form, a large solitary lesion can simulate an intracerebral tumor by exhibiting ring enhancement and restricted diffusion in its walls (26). However, key differentiators from tumors include the absence of magnetic susceptibility artifacts (hemorrhages) and low cerebral blood volume (CBV) and cerebral blood flow (CBF) values on perfusion maps (26, 27) (Figures 1, 2).

#### Aspergillosis

**Figure 3:**
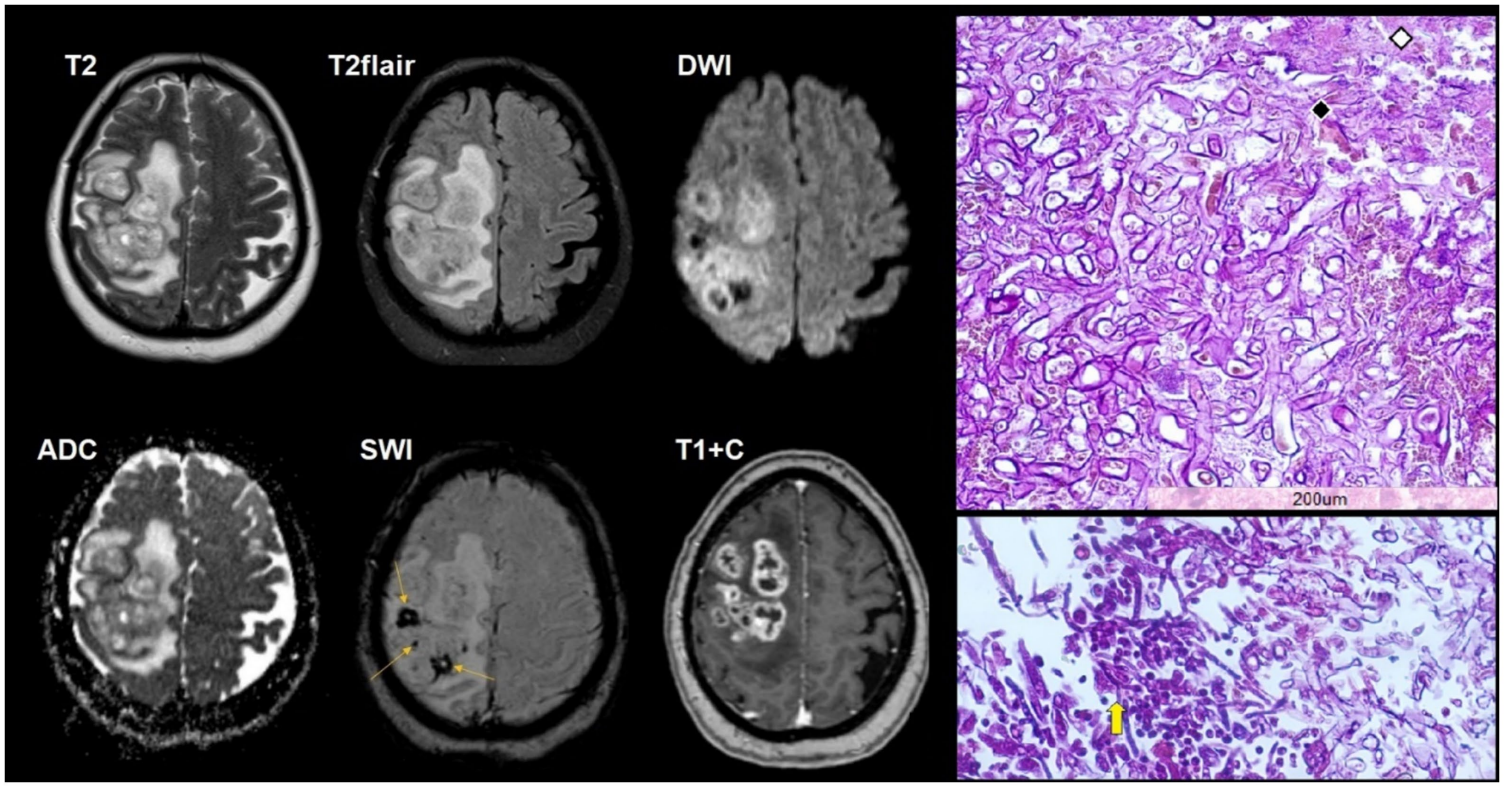
A 37-year-old HIV-positive woman, with cerebral aspergillosis, left-sided weakness and headaches, and disturbances in consciousness for 4 days. The CD4^+^ lymphocyte count was 80 cells/μL. Multiple ring-enhancing intra-axial lesions are present within the right frontoparietal white matter. The lesion walls show restricted diffusion, accompanied by moderate perifocal edema. Susceptibility artifacts are evident on SWI (yellow arrows). Microscopic characteristics: H&E staining; scale bar: 200 μm. Fungal hyphae permeate the vessel wall (black rhomb) with adjacent foci of hemorrhagic parenchymal infarction (white rhomb). Dichotomous, branching hyphae (yellow arrow) with Splendore-Hoeppli phenomenon (amorphous eosinophilic material around *Aspergillus*). Polymerase chain reaction (PCR) for *Aspergillus* species was positive.

**Figure 4:**
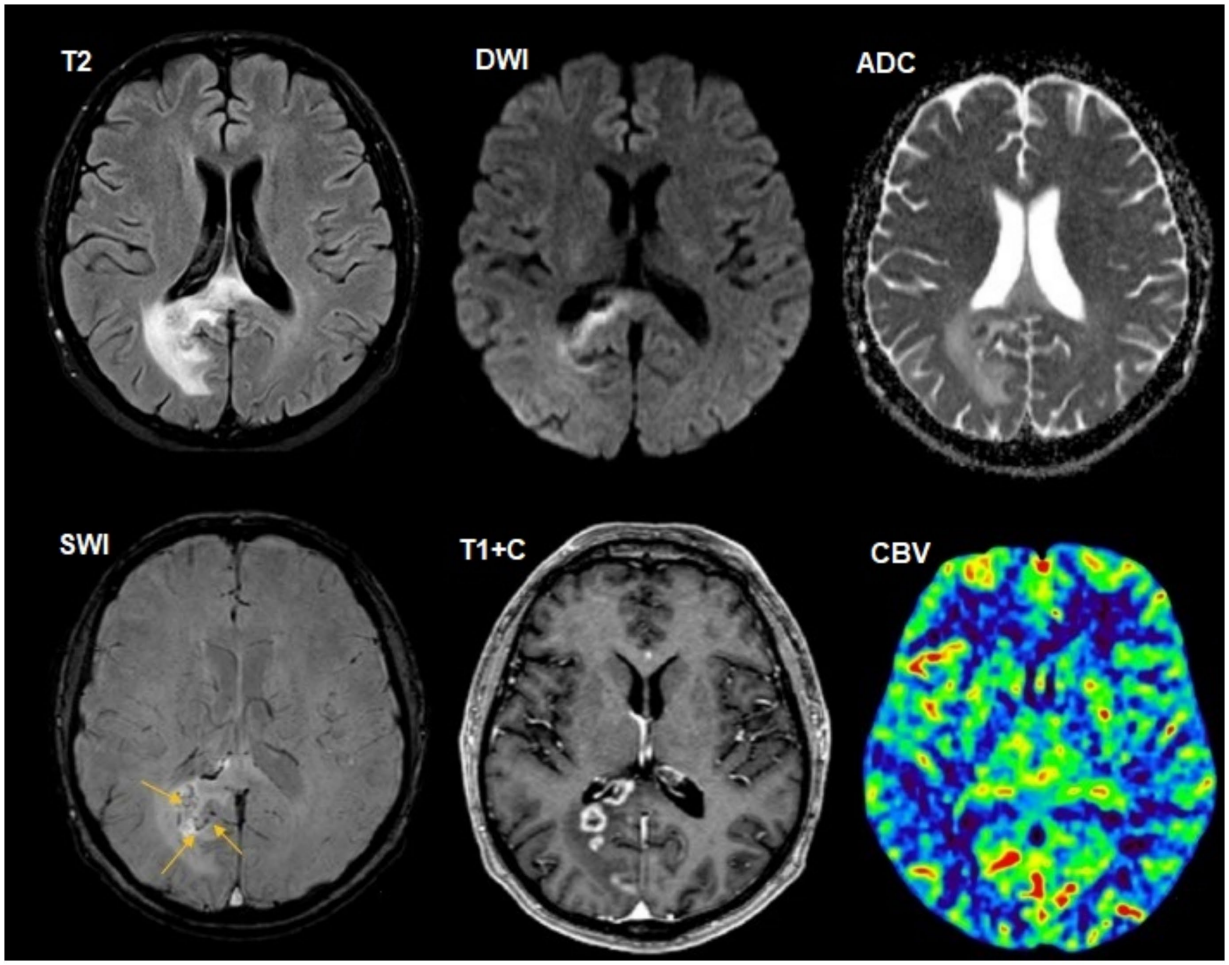
A 36-year-old HIV-positive male, with cerebral aspergillosis, dizziness, headache, and disturbances in consciousness for 1 week was included. The CD4^+^ lymphocyte count was 120 cells/μL. There was an intraaxial group of foci in the periventricular white matter of the right occipital lobe with a spread into the corpus callosum with ring-shaped contrast, restricted diffusion in the lesion walls, and perifocal edema. SWI indicates small foci of susceptibility artifacts (yellow arrows). DSC perfusion demonstrated low CBV values in the foci. Polymerase chain reaction (PCR) for *Aspergillus* species was positive.

Aspergillosis represents another serious opportunistic fungal infection in HIV-positive patients. This infection is etiologically linked to fungi of the genus *Aspergillu*s spp. and typically manifests in immunocompromised individuals (28). Brain involvement usually results from hematogenous dissemination from a primary focus in the lungs or sinuses (29). On imaging, neuroaspergillosis frequently manifests as multifocal abscesses exhibiting ring enhancement. Distinctive features include susceptibility artifacts on MRI images, due to the paramagnetic properties of fungal mycelia, and diffusion restrictions predominantly within the abscess walls (30, 31) (Figures 3, 4). Unlike tumors, these lesions also do not show increased perfusion levels.

#### Cryptocoссosis

Cryptococcosis is a fungal infection caused primarily by *Cryptococcus neoformans* in immunocompromised hosts (7, 32). After a primary lung infection occurs, the fungus spreads hematogenously to the CNS, especially when the CD4 + T-cell count is less than 50–100 cells/mm^3^ (7, 32).

**Figure 5:**
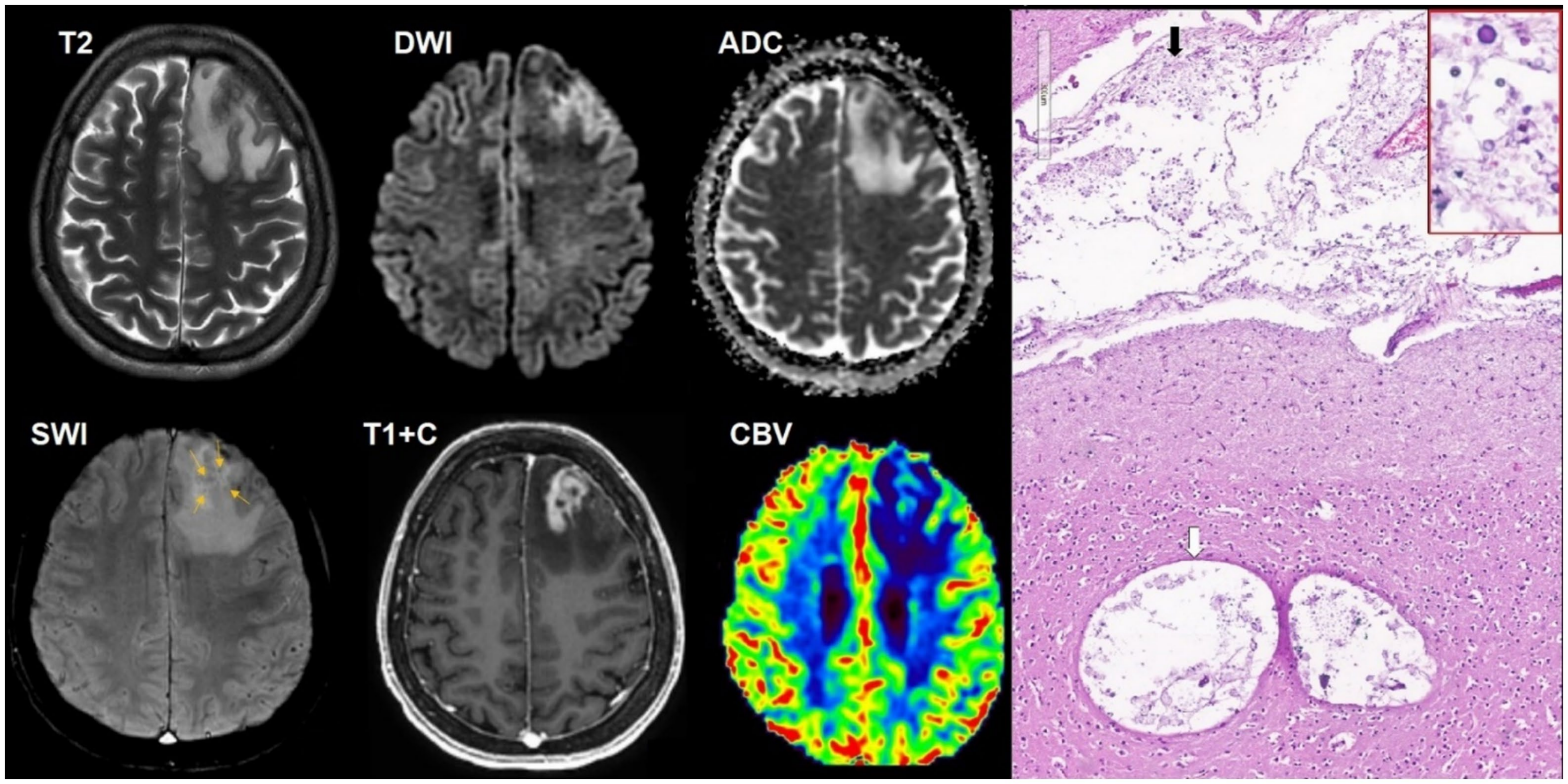
A 45-year-old HIV-positive male, with cerebral cryptococcosis with seizures. The CD4^+^ lymphocyte count was 80 cells/μL. Large intra-axial cryptococcoma with perifocal edema in the left frontal lobe with multilobulated peripheral enhancement and increased diffusion restriction. The patchy blooming of susceptibility sensitive sequences suggests the presence of blood products within the mass. There were no flow voids. DSC perfusion demonstrates low CBV values in the lesion. Microscopic characteristics: H&E staining scale bar: 300 μm. Red rectangle inset H&E staining, scale bar: 200 μm. Enlarged perivascular spaces filled with pale cryptococci give a spongy appearance to the brain tissue (white arrow); the inflammatory response is absent. Cryptococcal leptomeningitis with minimal macrophage infiltration (black arrow). Red rectangle: inconspicuous pale-stained encapsulated organisms of *Cryptococcus neoformans*. Polymerase chain reaction (PCR) for *Cryptococcus neoformans* was positive.

**Figure 6:**
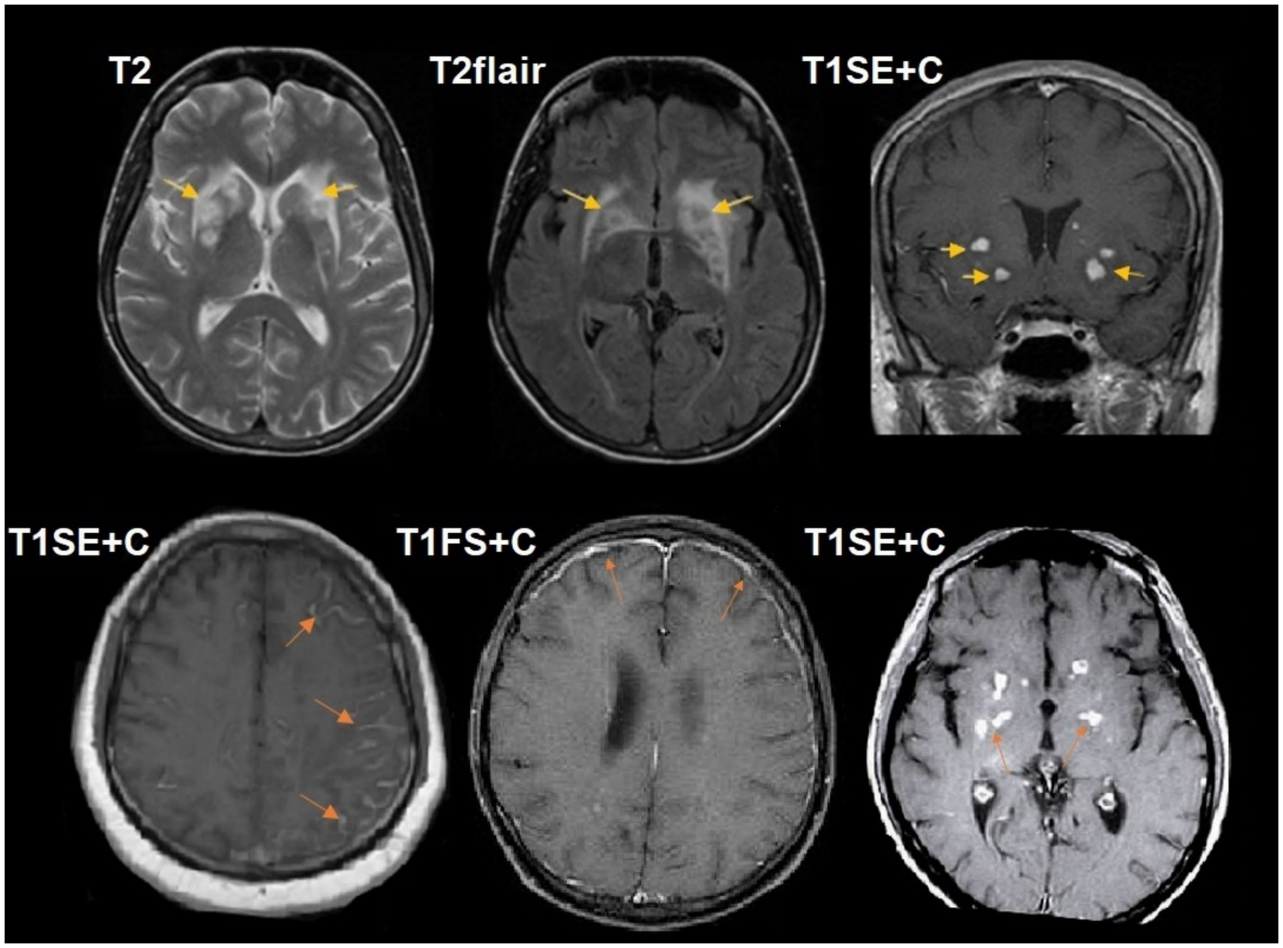
A 38-year-old HIV-positive male, with cerebral cryptococcosis, presented with altered sensorium and weakness. The CD4^+^ lymphocyte count was 85 cells/μL. Multiple, bilateral lesions in the basal ganglia, some of which are cystic in nature, with a left-sided predominance. Fronto-parietal leptomeningeal enhancement on the right and left sides, with small areas of associated parenchymal enhancement. Polymerase chain reaction (PCR) for *Cryptococcus neoformans* was positive.

The main CNS manifestations are meningitis, gelatinous pseudocysts, and cryptococcomas (33). Characteristic pseudocysts appear as clusters in the basal ganglia that retain signals on T2-FLAIR images (Figure 5). Other findings include leptomeningeal enhancement, secondary infarcts, and intraaxial cryptococcomas, which may show restricted diffusion in areas with high fungal concentrations (Figure 6) (7, 33–35). Magnetic resonance spectroscopy can aid in diagnosis by identifying trehalose, a biomarker for cryptococcal replication (36).

#### CNS-tuberculosis

**Figure 7:**
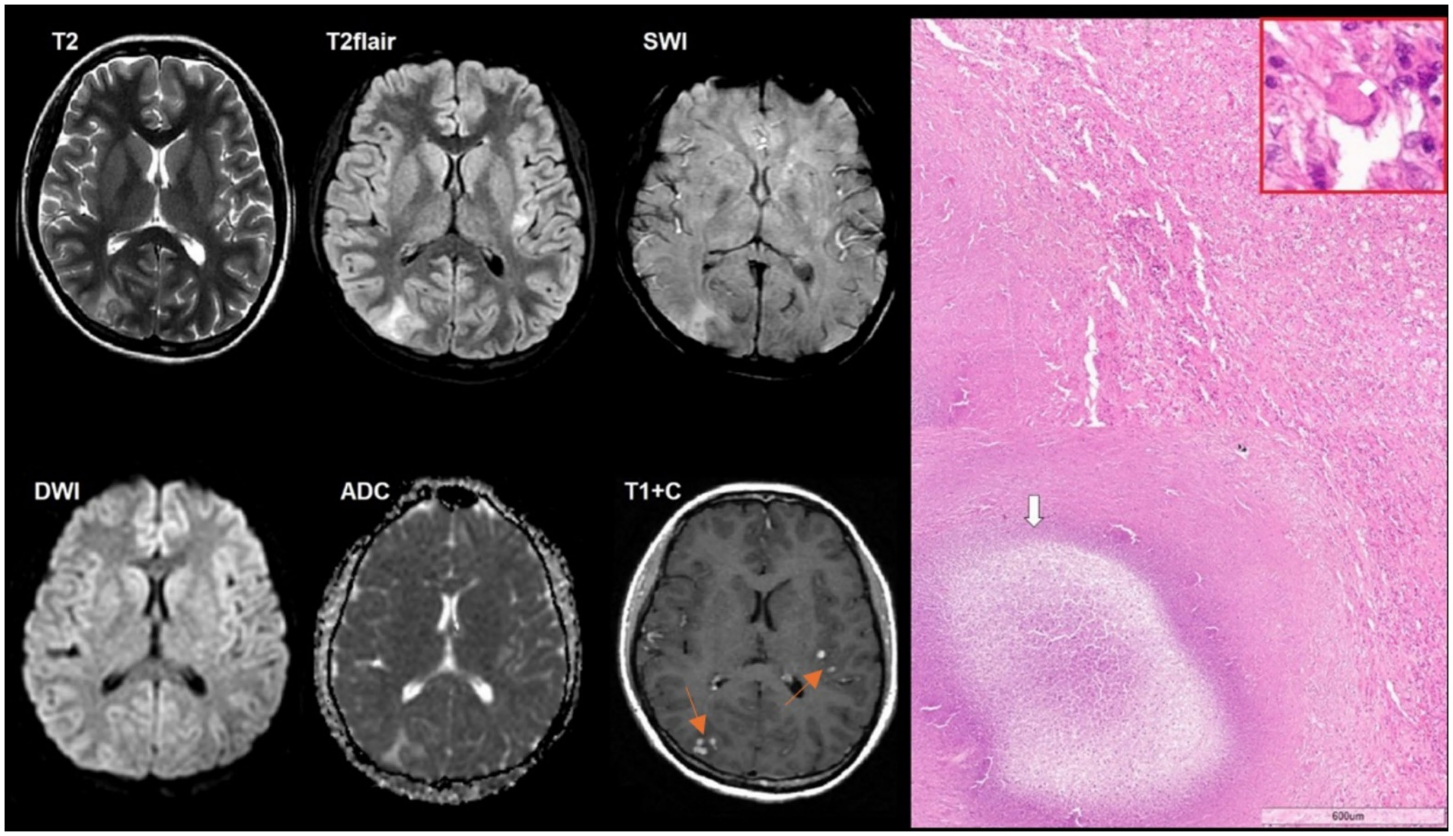
A 34-year-old HIV-positive male, with CNS tuberculosis and an acute altered mental status. The CD4^+^ lymphocyte count was 190 cells/μL. Several groups of intra-axial foci in the right occipital lobe and left subinsular region and mild perifocal edema with homogenous enhancement, without diffusion restriction were observed. SWI does not indicate susceptibility artifacts. Microscopic characteristics: H&E staining scale bar: 600 μm, red rectangle inset H&E staining scale bar: 200 μm. Tuberculoma (white arrow) with caseous central necrosis and surrounding epithelioid histiocytes, plasma cells, multinucleated cells, lymphocytes; perifocal brain tissue with reactive astrocytosis. Red rectangle: multinucleated giant cell Langhans type (white rhomb). Polymerase chain reaction (PCR) for *Mycobacterium tuberculosis* was positive.

CNS tuberculosis (CNS-TB) is a major coinfection in HIV patients and a significant cause of mortality (37, 38). It is diagnosed in up to 20% of tuberculosis cases in immunocompromised individuals (39). CNS-TB is postprimary form caused by *Mycobacterium tuberculosis* that hematogenously disseminates from the lungs to the brain (39, 40). Its radiological features include basilar leptomeningeal enhancement, bilateral basal ganglia infarctions from vasculitis, and parenchymal tuberculomas with either ring or homogeneous enhancement (Figure 7) (39, 41). These diverse imaging presentations require careful analysis to differentiate them from other infections and tumors.

### Demyelinating HIV-related lesions

HIV can also cause demyelinating lesions that mimic tumors, arising from autoimmune inflammation that destroys the myelin sheath. These lesions present in solitary, tumor-like (tumefactive demyelinating lesions), or multifocal (progressive multifocal leukoencephalopathy) forms (42, 43). Each type possesses distinct radiological features that are crucial for accurate identification. The following sections explore the specific imaging characteristics of these demyelinating conditions.

#### Tumefactive demyelinating lesions

**Figure 8:**
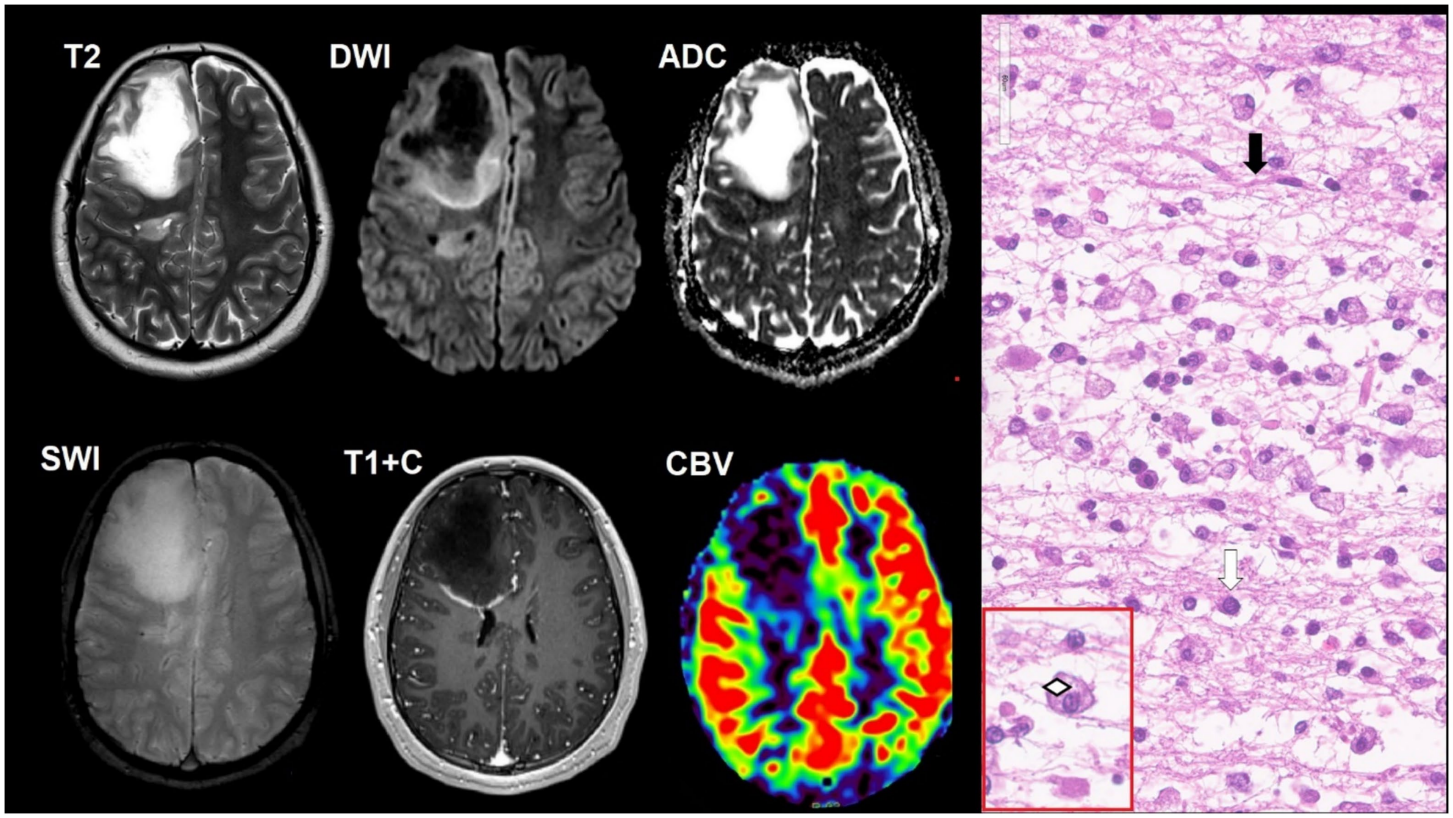
A 36-year-old HIV-positive woman, with a tumefactive demyelinating lesion with fever, aphasia, and weakness for 2 days was included. The CD4^+^ lymphocyte count was 200 cells/μL. There was an extensive intraaxial lesion in the right frontal lobe with open ring contrast enhancement, restricted diffusion in the peripheral regions, and mild perifocal edema. SWI does not indicate susceptibility artifacts. ASL perfusion demonstrates low CBF values in the lesion. Microscopic characteristics: H&E staining; scale bar: 60 μm, applies also for the inset in the red rectangle. Areas of myelin loss with reactive astrocytes (white arrow) with long tapering cytoplasmic processes (black arrow) and macrophage inflammation. Red rectangle: foamy macrophage (white rhomb).

Tumefactive demyelinating lesions (TDLs) are radiologically complex but have key features that distinguish them from tumors. These lesions typically show minimal mass effects, mild perilesional edema, a T2-hypointense rim, and an incomplete (open-ring) pattern of contrast enhancement. They exhibit restricted diffusion along their enhancing margins and demonstrate reduced CBV and CBF compared with those of normal brain tissue. Furthermore, their metabolic profile on MRS shows transient lipid/lactate peaks that resolve, followed by a marked reduction in NAA (43) (Figure 8). This combination of perfusion and metabolic findings is highly characteristic of demyelination.

#### Progressive multifocal leukoencephalopathy

**Figure 9:**
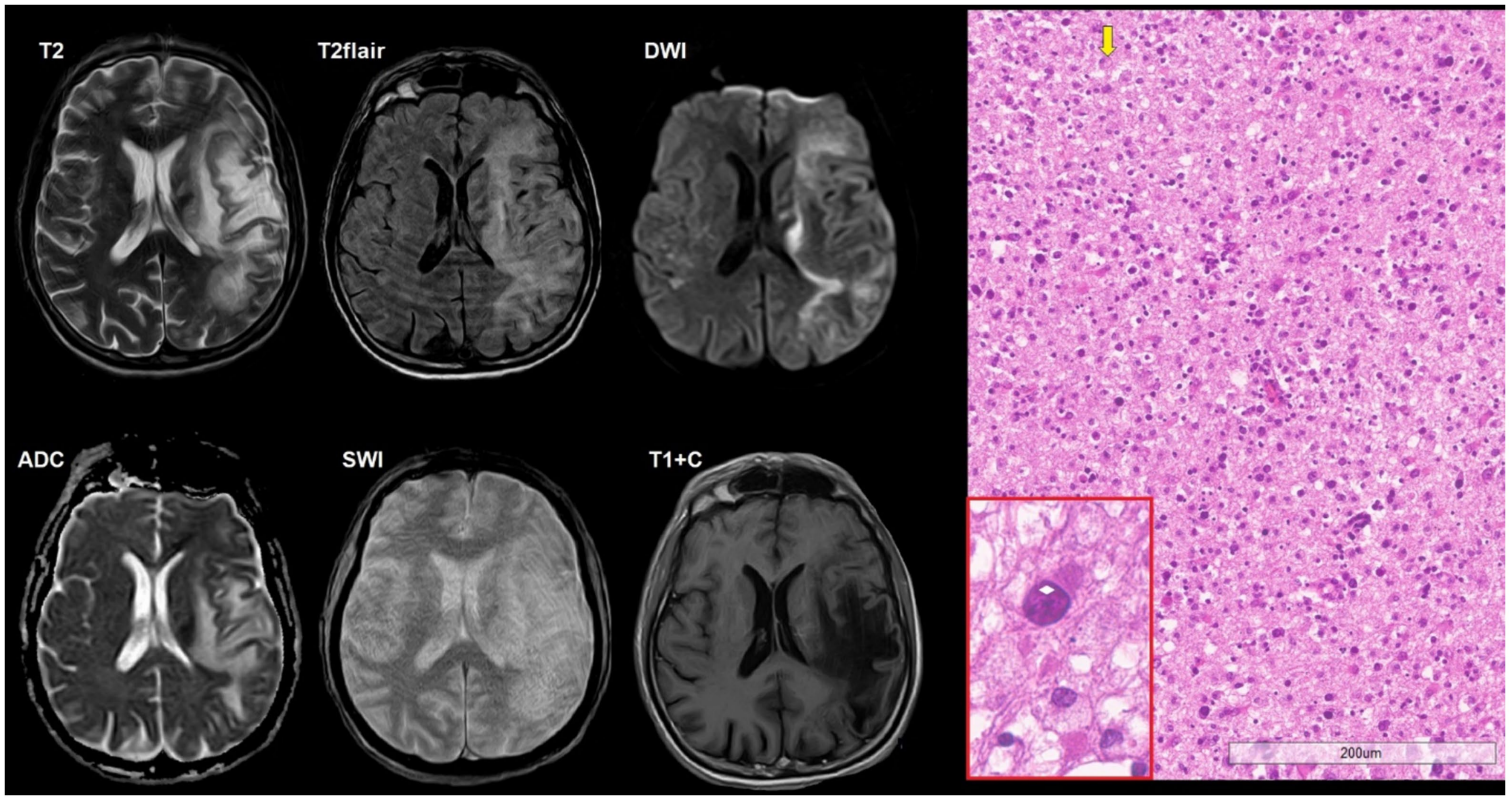
A 38-year-old HIV-positive male, with PML presented with cognitive dysfunction and motor weakness for 2 weeks. The CD4^+^ lymphocyte count was 40 cells/μL. There is an extensive area of subcortical and periventricular white matter lesions in the left cerebral hemisphere without contrast enhancement, with restricted diffusion along the lesion boundary. SWAN does not indicate susceptibility artifacts. Microscopic characteristics: H&E staining scale bar: 200 μm, red rectangle inset H&E staining scale bar: 60 μm. Numerous macrophages (yellow arrow) in areas of myelin loss, axonal injury, and limited lymphocytic infiltration; darkly stained nuclei of infected oligodendroglia and reactive astrocytosis. Red rectangle: viral inclusions in large round oligodendrocytes with hyperchromatic nuclei (white rhomb), without inclusions in the cytoplasm. Polymerase chain reaction (PCR) for DNA of the JC virus in cerebrospinal fluid was positive.

**Figure 10:**
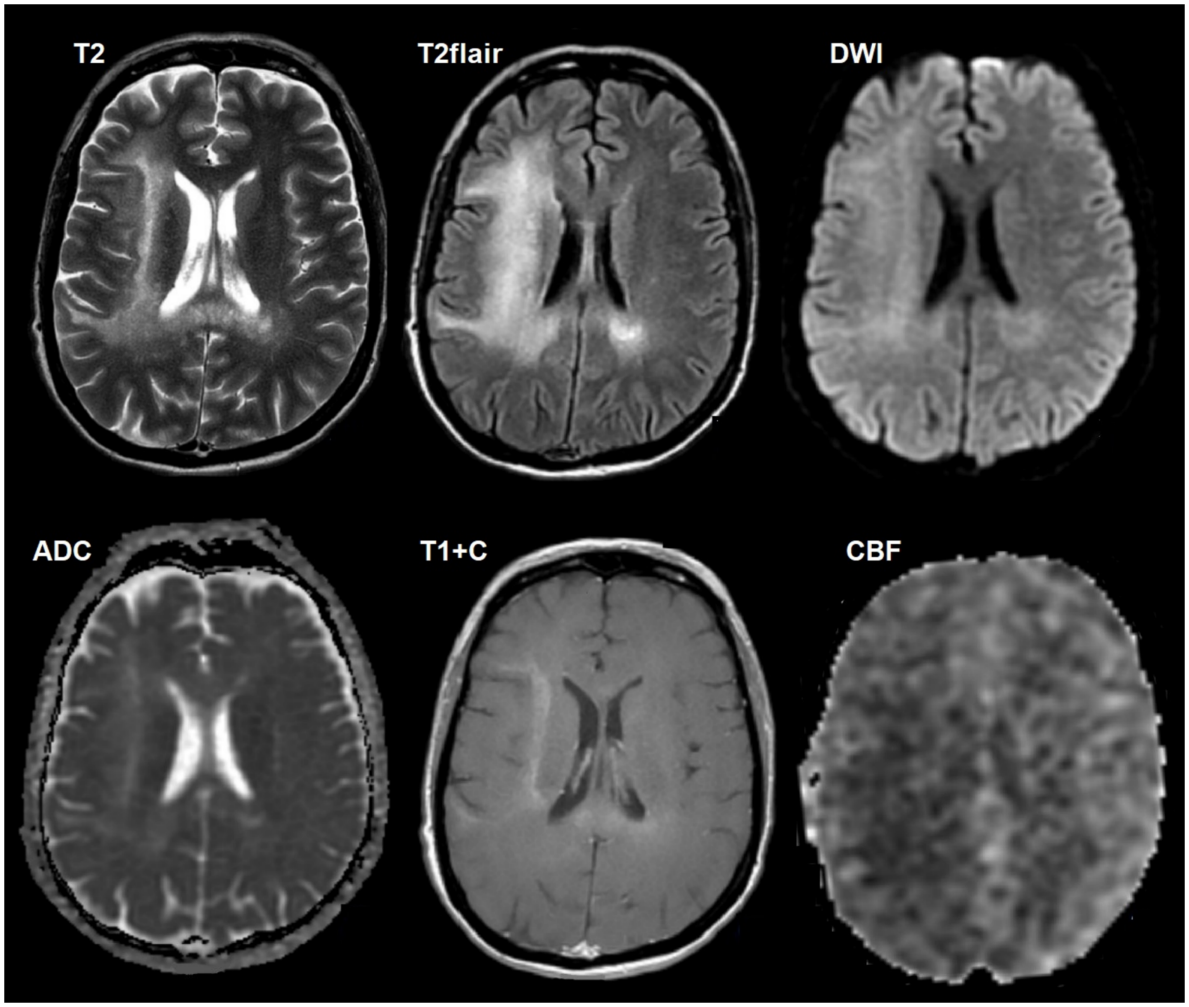
A 40-year-old HIV-positive male, with PML-IRIS with motor weakness and dysarthria for 1 month. The CD4^+^ lymphocyte count was 50 cells/μL. There is a unilateral lesion of the subcortical and periventricular white matter, mainly in the right cerebral hemisphere with contrast enhancing boundaries and facilitated diffusion. Raw ASL perfusion data revealed low CBF values in the lesion. Polymerase chain reaction (PCR) for DNA of JC virus in cerebrospinal fluid was positive.

Progressive multifocal leukoencephalopathy (PML) is a viral infection caused by the JC virus. It appears as multifocal, asymmetric areas affecting the subcortical white matter and U-fibers, often in the parieto-occipital region (44). The active areas of JC virus replication show restricted diffusion and a mild increase in relative CBV and CBF (45). Contrast enhancement is atypical unless it occurs in the context of immune reconstitution inflammatory syndrome (IRIS) or certain drug therapies, such as natalizumab (46). When present, enhancement at the lesion boundaries indicates increased BBB permeability (47) (Figures 9, 10). Recognizing the absence of typical tumor-like enhancement is a key diagnostic clue.

### Cerebrovascular disorders

**Figure 11:**
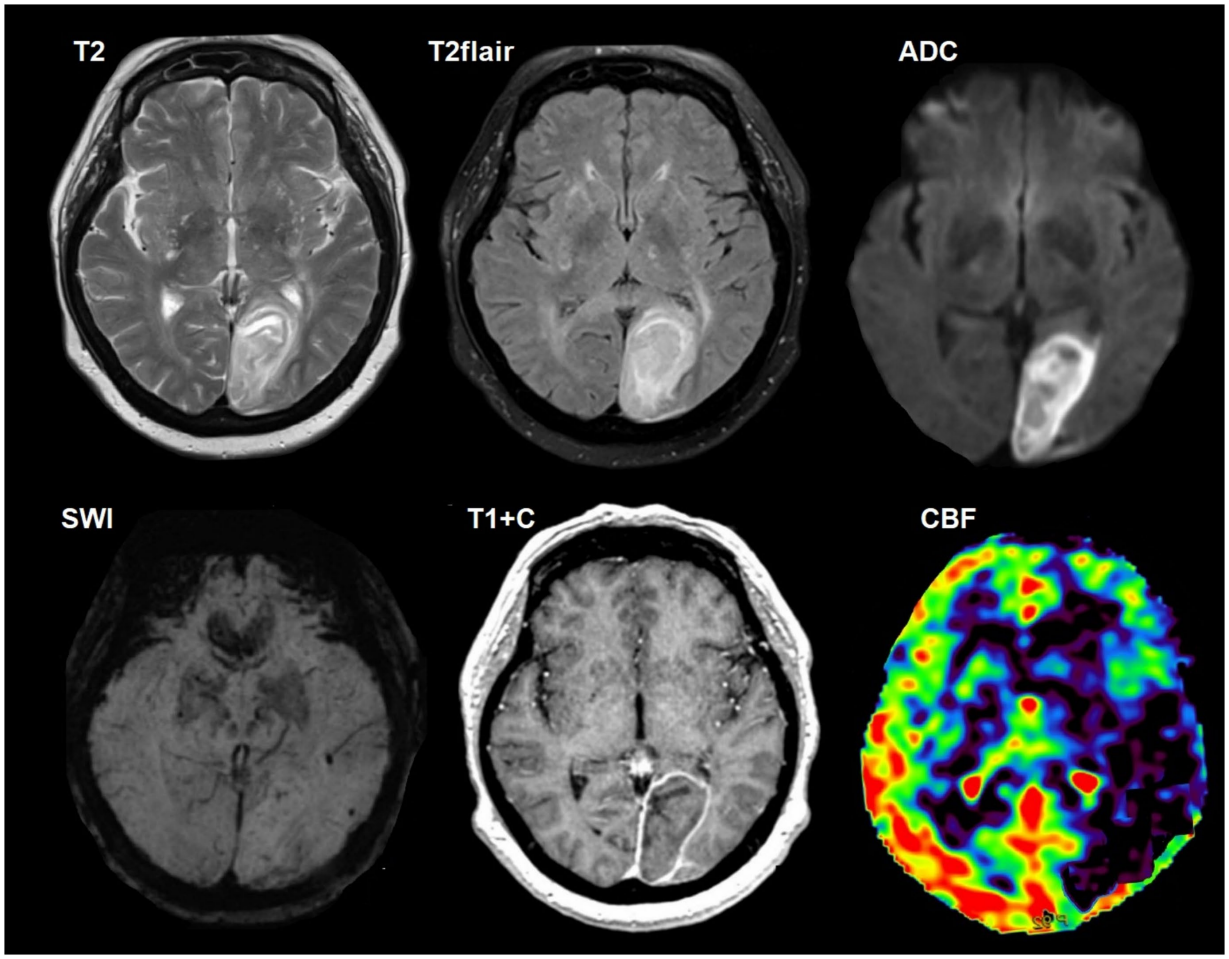
A 32-year-old HIV-positive woman, with cerebral infarction with right-sided weakness and hemianopsia for 3 days. CD4^+^ lymphocyte count was 150 cells/μL. There is a zone of cytotoxic edema in the left occipital lobe with ring-shaped contrast enhancement, mainly with gyral diffusion restriction. SWI revealed a linear artifact susceptibility in the P2 segment of the left posterior cerebral artery corresponding to a blood clot. ASL perfusion revealed low CBF values in the lesion as well as outside the left occipital lobe.

Cerebrovascular disease is a significant concern, as HIV infection drastically increases the risk of ischemic stroke. In patients with AIDS, the risk of ischemic stroke is nine times greater than that in the general population (48–50). The etiology is most often embolic, though vasculitis and opportunistic infections can also lead to cerebral infarctions (51–53, 65). Radiologically, strokes present with foci of cytotoxic edema and decreased perfusion within specific vascular territories. Pathological contrast enhancement is usually absent initially, but it can appear after the first week as a gyral pattern that must not be mistaken for a ring-enhancing tumor (52) (Figure 11). Understanding this evolution is essential to avoid a misdiagnosis of a neoplastic process.

### Tumors

#### Primary high grade glial tumors

**Figure 12:**
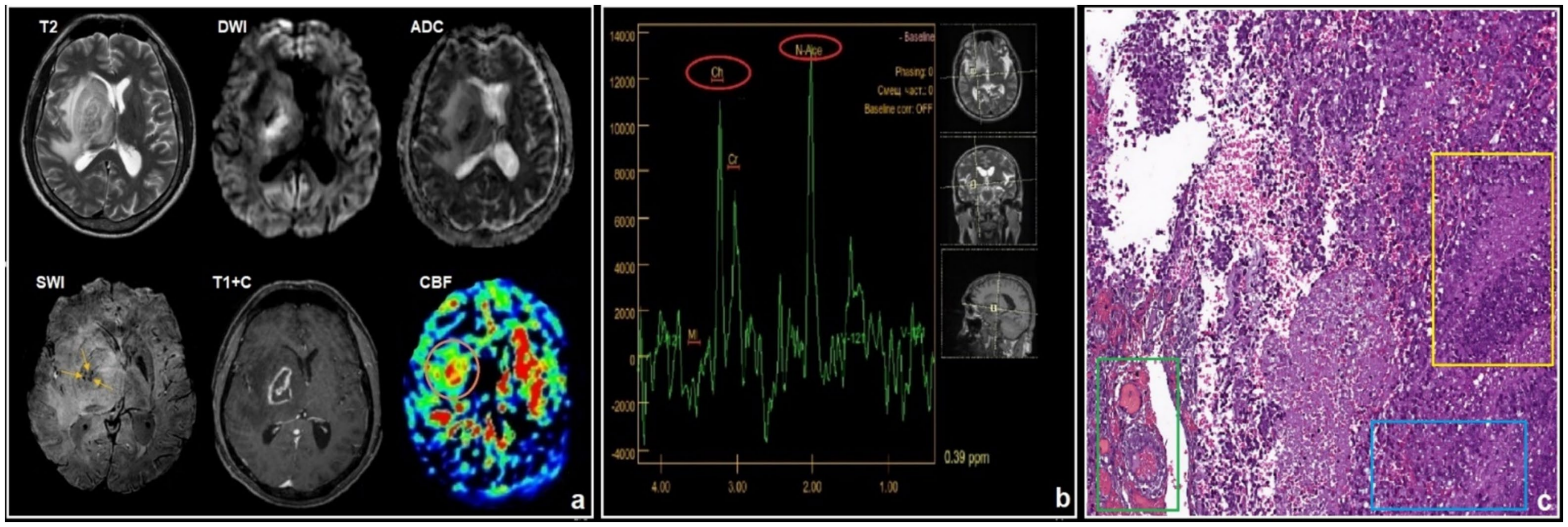
A 41-year-old HIV-positive male, with glioblastoma with acute complaints of weakness in the left extremities and disorientation. The CD4^+^ lymphocyte level count 180 cells/μL. The titer of antibodies to *T. gondii* was 1:400: **(a)** conventional MRI, **(b)** ^1^H-MRS, and **(c)** pathology. There is an intraaxial mass in the basal ganglia region on the right side, with ring-shaped contrast enhancement, restricted diffusion, and extensive perifocal edema. SWI revealed small foci of hemorrhages and vascular shunts (yellow arrows). ASL perfusion demonstrates high CBF values in the mass. MR spectroscopy revealed an elevated choline peak in the peripheral nonenhancement area. Microscopic characteristics: H&E staining scale bar: 40 μm. Heterogeneous tumor tissue comprised of atypical, polymorphic cells featuring hyperchromatic nuclei and an abundance of mitotic figures (blue rectangle); foci of necrosis surrounded by tumor cells with the formation of pseudopalisading structures (yellow rectangle); and cellular-vascular tumor area containing large vessels with prominent endothelial proliferation (green rectangle).

This paragraph details the classic and advanced imaging features of glioblastoma (GBM). A GBM typically appears as an intra-axial mass with ring-shaped contrast enhancement and a central necrotic core (Figure 12). GBM is surrounded by vasogenic edema and infiltrative components that show elevated choline on spectroscopy (54). The solid tumor portions show restricted diffusion, susceptibility artifacts from hemorrhage, and markedly increased relative CBV and CBF, often exceeding five times greater than those of the normal white matter (55). Furthermore, GBM displays pronounced T2 hyperintensity, a feature with a high diagnostic sensitivity of 97.1% that aids in differentiation (56). These combined advanced imaging characteristics are crucial for distinguishing GBM from other lesions.

#### Primary large B-cell lymphoma of the central nervous system

**Figure 13:**
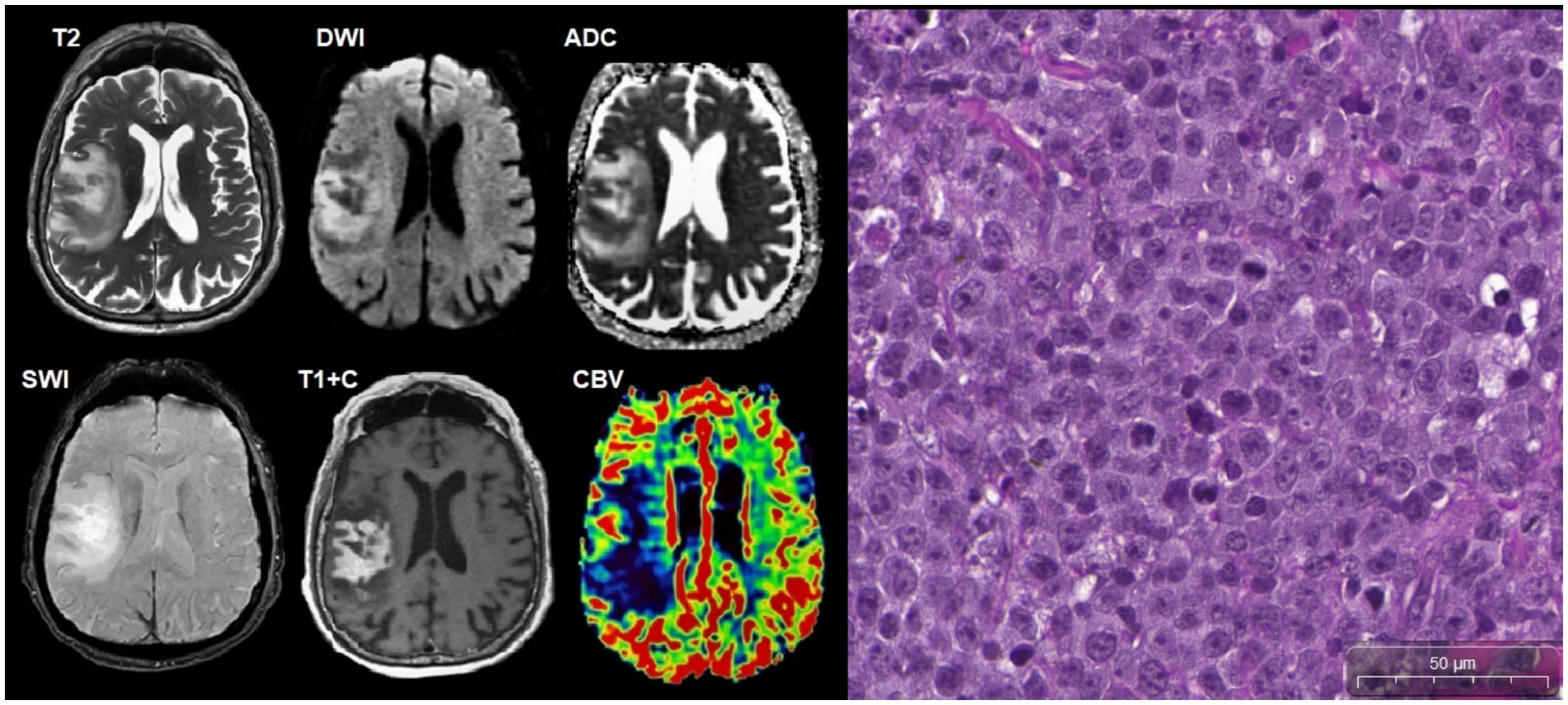
A 31-year-old HIV-positive woman, with classical PCNS-LBCL with dizziness, headaches, and left-sided weakness. The CD4^+^ lymphocyte count was 250 cells/μL. There was a tumor in the right frontal lobe with intense contrast enhancement, restricted diffusion, and moderate perifocal edema. SWI does not indicate susceptibility artifacts. DSC perfusion results in low CBV values in the mass. Microscopic characteristics: H&E staining scale bar: 50 μm. In hypercellular tumorous tissue with infiltrative growth, cells have a centroblast morphology, and multiple mitoses are present.

**Figure 14:**
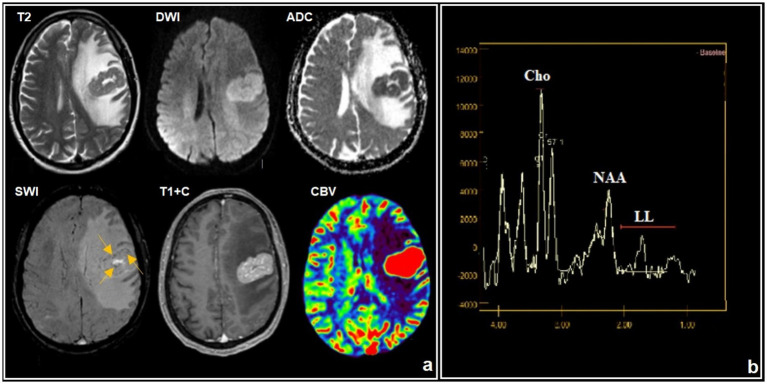
A 39-year-old HIV-positive male, with atypical PCNS-LBCL with right-sided weakness and disturbance in consciousness for 1 month. The CD4^+^ lymphocyte count was 180 cells/μL: **(a)** conventional MRI, **(b)** ^1^H-MRS. There is a tumor in the left frontal lobe with homogeneous contrast enhancement, restricted diffusion, and perifocal edema. SWI indicates small foci of susceptibility artifacts. DSC-perfusion demonstrates high CBV values in the mass that are greater than 5-fold. MR spectroscopy reveals an elevated choline peak with reduced NAA levels and a lipid lactate peak.

PCNS-LBCL is a thousandfold more common lymphoma in HIV-infected patients, occurring in approximately 10% of cases (7). The typical lesion is a solitary, periventricular or subpial mass that is T2 hypointense and shows marked diffusion restriction due to high cellularity. ADC ranges from 400 to 600 × 10^−6^ mm^2^/s (0.4–0.6 × 10^−3^ mm^2^/s) (55). The Ki-67 proliferation index is high in most cases. Perifocal edema is typically pronounced. Intratumoral calcifications and hemorrhagic foci are exceedingly rare and invisible even on SWI (57). A PCNS-LBCL lesion usually exhibits intense homogeneous enhancement with pronounced perifocal edema but rarely shows hemorrhage or calcification (Figure 13). In immunocompromised patients, lymphomas can appear atypically, mimicking glioma with ring enhancement, hemorrhage, and high perfusion (55, 57, 58) (Figure 14). In these challenging cases, magnetic resonance spectroscopy has become a valuable tool for identifying characteristic metabolic patterns. The pathognomonic markers of lymphomas are the lipid lactate peak and the elevated peak of choline within the tumor.

## Discussion

The present study demonstrates that while neoplastic and pseudotumoral CNS lesions in HIV-positive patients exhibit considerable morphological overlap on routine MRI (Table 1), their hemodynamic and metabolic profiles on multiparametric imaging provide distinctive diagnostic fingerprints. Our findings not only corroborate but also substantially expand previous observations regarding the pivotal role of mpMRI as a non-invasive tool for differential diagnosis in this complex patient population (59, 60).

**Table 1:** Comparative characteristics of tumoral and pseudotumoral lesions in the differential diagnosis of HIV-associated CNS pathologies.

mpMRI features	Tumor-like lesions						Tumors		
	Neurotoxoplasmosis/Aspergillosis	Cryptococcosis	CNS-tuberculosis	TDL	PML	Cerebral infarction	Glioblastoma	PCNSL	Atypical PCNSL
Preferential location	Variable	Basal ganglia, thalamus, and midbrain; basal cisterns (meningitis)	Basal ganglia (tuberculoma); basal cisterns (meningitis)	Supratentorial	Periventricular and subcortical with involving U-fibers	Corresponds to the affected vascular territories	Predominantly supratentorial	Periventricular and subpial	Periventricular and subpial
Type of lesion	Multiple, rarely solitary	Multiple, rarely large solitary	Multiple	Usually, a solitary large one	Multiple, asymmetric	Solitary or multiple	Solitary, less often multiple (multifocal/multicentric)	Usually solitary	Solitary or multiple
Edema	Moderate vasogenic	Absent; moderate vasogenic (cryptococcoma)	Mild vasogenic	Moderate vasogenic or absent	Absent	Cytotoxic	Severe vasogenic	Moderate vasogenic	Moderate vasogenic
Contrast enhancement (CE) on T1-W	Nodular or ring CE	Variable, often no CE (cryptococcoma); lepto-pachymeningeal CE (meningitis)	Ring-shaped (tuberculoma and abscess) and homogenous (tuberculoma); lepto-pachymeningeal CE (meningitis)	Open-ring CE	Absent, rarely patchy or punctate CE at the lesion border in IRIS	Gyral CE, rarely ring CE	Ring CE	Homogenic vivid CE	Homogenic vivid CE, sometimes ring CE
Hemorrhage	Typical for fungal etiology	Absent	Absent	Not typical	Not typical	Present in cases of hemorrhagic transformation	Always present	Absent	Sometimes occur
DWI/ADC	Restricted in the wall	Variable (cryptococcoma); significant restricted in infarcts	Significant restricted in abscess cavity and infarcts	Restricted on the periphery, facilitated in the center	Restricted at the lesion border	Significant restricted	Restricted in the solid component	Significant restricted	Significant restricted
DSC/ASL	Low	Low	Low	Variable, usually low	High at the lesion border	Low	Always high	Low	Often high

A key finding of our analysis is the significant overlap in imaging semiotics between neoplastic and pseudotumoral conditions. GBM, atypical lymphoma, TDL, and PML may all present with T2-hyperintensity and ill-defined contrast enhancement. We posit that this similarity is attributable to shared pathophysiological mechanisms operating under conditions of profound immunosuppression. Chronic HIV-related neuroinflammation, characterized by microglial activation and subsequent increased permeability of the BBB, creates a permissive environment for vasogenic edema and contrast leakage, irrespective of the underlying etiology (61). This underscores the limitation of relying solely on conventional sequences in this cohort.

In contrast to studies primarily focused on nosological specificity, our analysis emphasizes the correlation between CBV and intratumoral heterogeneity. We confirm the findings of Batalov et al. (62) that maximally elevated CBV, CBF serves as a robust marker for GBM. However, we further refine this observation by demonstrating that within a comparative framework, elevated CBV correlates not merely with the presence of a tumor but specifically with its morphological heterogeneity—namely, necrosis and endothelial proliferation. This distinguishes GBM from primary CNS lymphoma, where CBV elevation tends to be more homogeneous and strictly confined to areas of active contrast enhancement (55).

Our data align with those of Feng et al. (63), who reported that elevated choline peak in perifocal areas is more specific to glial tumors than to lymphomas, given the less infiltrative growth pattern of the latter. Nevertheless, we observed that in select cases of inflammatory PML (i.e., PML-IRIS), a moderate choline peak may also be present, creating a diagnostic “gray zone.” In such ambiguous cases, perfusion analysis proves decisive: PML typically lacks significant CBV elevation, reflecting the absence of neoangiogenesis, in stark contrast to GBM.

The confirmed specificity of imaging signs for cerebral toxoplasmosis (peripheral diffusion restriction) and aspergillosis (magnetic susceptibility artifacts on SWI) has a clear pathomorphological basis. The toxoplasmic abscess forms a dense capsule containing viable bradyzoites at the periphery, explaining the rim of restricted diffusion. Conversely, susceptibility artifacts in aspergillosis, as rightly noted by Frascheri et al. (30), result from hemorrhages, fungal hyphae, and calcifications—hallmarks of angioinvasive fungal growth.

Our observation that cryptococcosis and tuberculosis each manifest in three primary forms (meningitis, parenchymal lesions, and pseudocysts/abscesses) elucidates the high rate of diagnostic errors when routine protocols are used. Unlike studies that examine these entities in isolation, our comparative analysis highlights the particular difficulty in differentiating gelatinous pseudocysts of cryptococcosis from small caseating tuberculomas. In such challenging scenarios, analysis of the specific cerebral structures involved may serve as an additional discriminating criterion. For instance, cryptococcal pseudocysts typically involve dilated perivascular spaces around the basal ganglia, whereas tuberculomas preferentially localize at the corticomedullary junction.

The substantial polymorphism confirmed in our study directly correlates with the findings of large multicenter trials, which report a high rate of empirical therapy initiation in HIV-positive patients. We concur with Kizaki et al. (64) that, in cases of unclear etiology, brain biopsy remains indispensable for verifying the diagnosis to guide targeted therapy and improve outcomes. However, given the high invasiveness and inherent risks of this procedure in immunocompromised individuals, the pursuit of reliable non-invasive alternatives is imperative.

Our study demonstrates that integrating morphological features with PWI and ^1^H-MRS data can overcome the inherent limitations of standard MRI. This integrated approach not only confirms the high specificity of mpMRI reported in the literature but also refines the differentiation criteria in the most diagnostically complex situations. By combining perfusion data, spectroscopic metabolites, and morphological patterns, we can significantly reduce diagnostic errors in immunocompromised patients.

### Limitations

Currently, MRI perfusion methods are widely used in clinical practice. However, it should be recognized that not all institutions correctly perform perfusion parameter measurements (CBVs, СBFs) according to a standardized region of interest (ROI) analysis method. The assessment is often carried out subjectively, on the basis of visual interpretation of color maps, which can lead to significant measurement inaccuracies and, as a result, errors in diagnostic conclusions.

MRS also has technical constraints, including low sensitivity, poor spatial resolution causing partial volume effects, and artifacts from lipids or magnetic susceptibility. Furthermore, MRS is time-consuming, and often requires 10–15 min per area, which increases the number of scans and patient discomfort.

Confirmatory testing also faces hurdles, as many infections need PCR for diagnosis owing to difficulties with H&E staining interpretation without special stains. While useful, immunohistochemistry is expensive and not universally available. In burn-out cases, it is not possible to detect toxoplasmosis or JC virus in biopsy. This may lead to the use of PCR for cerebrospinal fluid. Sometimes, a brain biopsy under general anesthesia is ultimately needed, as in cases of misdiagnosed glioma.

To minimize the need for invasive diagnostic procedures, particularly brain biopsy, it is necessary to develop and implement standardized quantitative protocols for perfusion MRI analysis, actively employ radiomic techniques, and advance MR spectroscopy technologies by increasing their resolution and reducing acquisition times. The integration of these improved approaches, combined with clinical data and more accessible molecular testing will create a more accurate and safer diagnostic algorithm for patients.

In our clinical experience, the use of even the most comprehensive atlases, tutorials and image galleries has diagnostic limitations. Confidence in confirming a pathological process can be achieved only in the presence of specific and unique imaging patterns for a particular type of disease. In the absence of these patterns or when the visual characteristics of different diseases overlap, significant difficulties in differential diagnosis arise. This often narrows the diagnostic considerations to the dilemma of “tumor vs. neuroinfection.”

## Conclusion

Noninvasive verification of neoplastic and pseudotumoral lesions in HIV-positive patients presents a major diagnostic challenge for achieving early characterization. These neoplastic and pseudotumoral lesions often share substantial similarities on conventional neuroimaging. However, unique diagnostic markers from mpMRI mapping can facilitate the determination of the etiology of the pathological process. This approach is especially valuable in clinical settings with limited historical and examination data. Therefore, a systematic mpMRI protocol is essential for accurate analysis.

The list of possible diagnoses we presented is not exhaustive, but it covers the most critical nosological forms. The specific detailed neuroimaging characteristics provide a solid foundation for refining the differential diagnosis. Radiologists should always consider both neoplastic and pseudotumoral processes in patients with weakened immunity. Using a standardized mpMRI protocol and a comparative table of characteristics will guide a more structured and accurate diagnostic algorithm.

Mastering the interpretation of these mpMRI findings allows radiologists to assume a key role in the clinical team. This knowledge enables timely and accurate diagnosis of cerebral lesions in HIV-infected patients. Consequently, radiologists directly contribute to selecting the correct treatment pathway and improving clinical outcomes. Their expertise in advanced imaging is therefore indispensable for managing this complex patient population.

Future research should focus on conducting prospective multicenter studies incorporating standardized scanning protocols and the use of radiomics techniques. Implementation of this approach will enable the development of highly accurate predictive mathematical models, which, in turn, will help reduce clinical practice’s dependence on invasive diagnostic procedures and facilitate the timely initiation of targeted therapy in patients with HIV infection.

## Glossary

ADC: Apparent diffusion coefficient
AIDS: Acquired immunodeficiency syndrome
ASL: Arterial spin labeling
BBB: Blood–brain barrier
CBF: Cerebral blood flow
CBV: Cerebral blood volume
CNS: Central nervous system
CNS-TB: CNS tuberculosis
DWI: Diffusion-weighted imaging
DSC: Dynamic susceptibility contrast
GBM: Glioblastoma
H&E: Hematoxylin and eosin
HIV: Human immunodeficiency virus
IRIS: Immune reconstitution inflammatory syndrome
JC virus: John Cunningham virus
mpMRI: Multiparametric magnetic resonance imaging
MRI: Magnetic resonance imaging
PCNS-LBCL: Primary central nervous system large B-cell lymphoma
PCR: Polymerase chain reaction
PML: Progressive multifocal leukoencephalopathy
PWI: Perfusion-weighted imaging
ROI: Region of interest
SWI: Susceptibility-weighted imaging
TDLs: Tumefactive demyelinating lesions
WHO: World Health Organization
^1^H-MRS: Proton magnetic resonance spectroscopy

## References

[1] Swinkels HM Justiz Vaillant AA Nguyen AD Gulick PG. "HIV and AIDS". In: StatPearls. Treasure Island, FL: StatPearls Publishing (2024)

[2] Genowska A Zarębska-Michaluk D Parczewski M Strukcinskiene B Rzymski P Flisiak R. Impact of the COVID-19 and war migration on HIV/AIDS epidemiology in Poland. J Clin Med. (2024) 13:4106. doi: 10.3390/jcm13144106, 39064146 PMC11278201

[3] Justiz Vaillant AA Naik R. "HIV-1—associated opportunistic infections". In: StatPearls. Treasure Island, FL: StatPearls Publishing (2023)

[4] Meng S Tang Q Xie Z Wu N Qin Y Chen R . Spectrum and mortality of opportunistic infections among HIV/AIDS patients in southwestern China. Eur J Clin Microbiol Infect Dis. (2023) 42:113–20. doi: 10.1007/s10096-022-04528-y, 36413338 PMC9816182

[5] de Melo SA Pinto SD Ferreira EDS Brotas R Marinho EPM da Silva VA . Molecular diagnosis of opportunistic infections in the central nervous system of HIV-infected adults in Manaus, Amazonas. Front Med. (2024) 10:1298435. doi: 10.3389/fmed.2023.1298435, 38264048 PMC10803427

[6] Uwishema O Ayoub G Badri R Onyeaka H Berjaoui C Karabulut E . Neurological disorders in HIV: Hope despite challenges. Immun Inflamm Dis. (2022) 10:e591. doi: 10.1002/iid3.591, 35146953 PMC8926501

[7] Sakai M Higashi M Fujiwara T Uehira T Shirasaka T Nakanishi K . MRI imaging features of HIV-related central nervous system diseases: diagnosis by pattern recognition in daily practice. Jpn J Radiol. (2021) 39:1023–38. doi: 10.1007/s11604-021-01150-4, 34125369 PMC8202053

[8] Hanna KF Sayles HR O’Neill J White ML Wilson TW Swindells S. Incidental findings on brain MRI in people with HIV infection. Sci Rep. (2020) 10:9474. doi: 10.1038/s41598-020-66443-6, 32528044 PMC7289834

[9] Torres YC Alves-Leon SV Lima MA. Frequency of pseudotumoral central nervous system lesions in an oncology center. World Neurosurg. (2019) 130:e333–7. doi: 10.1016/j.wneu.2019.06.083, 31228702

[10] Alleg M Solis M Baloglu S Cotton F Kerschen P Bourre B . Progressive multifocal leukoencephalopathy: MRI findings in HIV-infected patients are closer to rituximab- than natalizumab-associated PML. Eur Radiol. (2021) 31:2944–55. doi: 10.1007/s00330-020-07362-y, 33155106 PMC7644389

[11] Ding X Liang T Liang B Zhou X Chen J Gao H . Clinical characteristics and prognostic analysis of patients with HIV and glioma: a case series and literature review. Exp Ther Med. (2024) 27:90. doi: 10.3892/etm.2024.12380, 38274346 PMC10809446

[12] Patel N Charate R. Diagnosis of primary CNS lymphoma in a HIV patient with multiple ring-enhancing lesions. IDCases. (2021) 24:e01065. doi: 10.1016/j.idcr.2021.e01065, 33850716 PMC8022152

[13] Zedde M Napoli M Moratti C Pavone C Bonacini L di Cecco G . Tumor-like lesions in primary angiitis of the central nervous system: the role of magnetic resonance imaging in differential diagnosis. Diagnostics. (2024) 14:618. doi: 10.3390/diagnostics14060618, 38535038 PMC10969781

[14] Perez Giraldo GS Singer L Cao T Jamshidi P Dixit K Kontzialis M . Differential diagnosis of tumor-like brain lesions. Neurol Clin Pract. (2023) 13:e200182. doi: 10.1212/CPJ.0000000000200182, 37664132 PMC10468256

[15] Kwon YM Han SH Sung KS Song YJ. Cerebral microangiopathy mimicking a high-grade glioma in old age: a case report. Brain Tumor Res Treat. (2022) 10:195–9. doi: 10.14791/btrt.2022.0021, 35929118 PMC9353164

[16] D’Antonio F Spinello Z Bargiacchi L Splendiani E Rossi S Masuelli L . Circulating microRNAs: a remarkable opportunity as non-invasive biomarkers from adult to pediatric brain tumor patients. Crit Rev Oncol Hematol. (2025) 208:104650–8428. doi: 10.1016/j.critrevonc.2025.104650, 39914569

[17] Piwowarek M Siennicka K Mikuła T Wiercińska-Drapało A. Cerebral toxoplasmosis, CMV and bacterial pneumonia with decreasing CD4^+^ T-cell count as results of antiretroviral therapy discontinuation—a case report. Pathogens. (2021) 10:497. doi: 10.3390/pathogens10040497, 33924043 PMC8073605

[18] Wu W Li J Ye J Wang Q Zhang W Xu S. Differentiation of glioma mimicking encephalitis and encephalitis using multiparametric MR-based deep learning. Front Oncol. (2021) 11:639062. doi: 10.3389/fonc.2021.639062, 33791225 PMC8005708

[19] Sanvito F Kaufmann TJ Cloughesy TF Wen PY Ellingson BM. Standardized brain tumor imaging protocols for clinical trials: current recommendations and tips for integration. Front Radiol. (2023) 3:1267615. doi: 10.3389/fradi.2023.1267615, 38152383 PMC10751345

[20] Ellingson BM Bendszus M Boxerman J Barboriak D Erickson BJ Smits M . Jumpstarting brain tumor drug development coalition imaging standardization steering committee. Consensus recommendations for a standardized brain tumor imaging protocol in clinical trials. Neuro-Oncology. (2015) 17:1188–98. doi: 10.1093/neuonc/nov095, 26250565 PMC4588759

[21] Kaufmann TJ Smits M Boxerman J Huang R Barboriak DP Weller M . Consensus recommendations for a standardized brain tumor imaging protocol for clinical trials in brain metastases. Neuro-Oncology. (2020) 22:757–72. doi: 10.1093/neuonc/noaa030, 32048719 PMC7283031

[22] Leu K Boxerman JL Ellingson BM. Effects of MRI protocol parameters, preload injection dose, fractionation strategies, and leakage correction algorithms on the fidelity of dynamic-susceptibility contrast MRI estimates of relative cerebral blood volume in gliomas. AJNR Am J Neuroradiol. (2017) 38:478–84. doi: 10.3174/ajnr.A5027, 28034995 PMC7959993

[23] Boxerman JL Prah DE Paulson ES Machan JT Bedekar D Schmainda KM. The role of preload and leakage correction in gadolinium-based cerebral blood volume estimation determined by comparison with MION as a criterion standard. AJNR Am J Neuroradiol. (2012) 33:1081–7. doi: 10.3174/ajnr.A2934, 22322605 PMC4331024

[24] van Dorth D Venugopal K van der Werff KN Smits M Warnert EAH Hernandez-Tamames JA . Exploring the need for a preload on the estimation of permeability, vessel radius, and relative cerebral blood volume in MR vascular fingerprinting-based dynamic susceptibility contrast perfusion imaging. Magn Reson Med. (2025) 93:1761–70. doi: 10.1002/mrm.30383, 39607907 PMC11782708

[25] Ayoade F Joel Chandranesan AS. "HIV-1—associated toxoplasmosis". In: StatPearls. Treasure Island, FL: StatPearls Publishing (2022) 28722907

[26] Ng CF Chong CY. Cerebral toxoplasmosis in systemic lupus erythematosus. Neurohospitalist. (2021) 11:377–8. doi: 10.1177/19418744211005324, 34567404 PMC8442163

[27] Marcus C Feizi P Hogg J Summerfield H Castellani R Sriwastava S . Imaging in differentiating cerebral toxoplasmosis and primary CNS lymphoma with special focus on FDG PET/CT. AJR Am J Roentgenol. (2021) 216:157–64. doi: 10.2214/AJR.19.22629, 33112669

[28] Diallo ID Lrhorfi N Traoré WM Onka B Chat L Allali N. Neurological involvement complicating invasive pulmonary aspergillosis. Glob Pediatr Health. (2022) 9:2333794X221098829. doi: 10.1177/2333794X221098829, 35614911 PMC9125102

[29] Amanati A Lotfi M Abdolkarimi B Karimi Rouzbahani A Mahmoudvand G. Evolution of neuroimaging findings in angioinvasive cerebral aspergillosis in a pediatric patient with leukemia during long-term observation. BMC Infect Dis. (2023) 23:811. doi: 10.1186/s12879-023-08483-7, 37978456 PMC10657136

[30] Frascheri L Breivik B Helleren R. Cerebral aspergillose. Tidsskr Nor Laegeforen. (2021) 141. doi: 10.4045/tidsskr.20.0825 33876620

[31] Finelli PF. MR target sign in cerebral aspergillosis. Neurohospitalist. (2020) 10:287–90. doi: 10.1177/1941874420929191, 32983348 PMC7495708

[32] Dao A Kim HY Garnham K Kidd S Sati H Perfect J . Cryptococcosis—a systematic review to inform the World Health Organization fungal priority pathogens list. Med Mycol. (2024) 62:myae043. doi: 10.1093/mmy/myae043, 38935902 PMC11210623

[33] Anjum SH Bennett JE Dean O Marr KA Hammoud DA Williamson PR. Neuroimaging of cryptococcal meningitis in patients without human immunodeficiency virus: data from a multi-center cohort study. J Fungi. (2023) 9:594. doi: 10.3390/jof9050594, 37233305 PMC10220536

[34] Hu Z Luo H Hu D Peng Y. Neuroimaging findings of disseminated cryptococcosis in children and correlation with prognosis. Pediatr Radiol. (2025) 55:1515–25. doi: 10.1007/s00247-025-06277-4, 40464909

[35] Brunasso L Costanzo R Cascio A Florena A Sparacia G Iacopino DG . Seizure in isolated brain cryptococcoma: case report and review of the literature. Surg Neurol Int. (2021) 12:153. doi: 10.25259/SNI_805_2020, 33948323 PMC8088491

[36] Roosen L Maes D Musetta L Himmelreich U. Preclinical models for cryptococcosis of the CNS and their characterization using in vivo imaging techniques. J Fungi. (2024) 10:146. doi: 10.3390/jof10020146, 38392818 PMC10890286

[37] GBD 2019 Tuberculosis Collaborators. Global, regional, and national sex differences in the global burden of tuberculosis by HIV status, 1990–2019: results from the Global Burden of Disease Study 2019. Lancet Infect Dis. (2022) 22:222–41. doi: 10.1016/S1473-3099(21)00449-7 34563275 PMC8799634

[38] Nosik M Ryzhov K Kudryavtseva AV Kuimova U Kravtchenko A Sobkin A . Decreased IL-1 β secretion as a potential predictor of tuberculosis recurrence in individuals diagnosed with HIV. Biomedicine. (2024) 12:954. doi: 10.3390/biomedicines12050954, 38790916 PMC11117744

[39] Dahal P Parajuli S. Magnetic resonance imaging findings in central nervous system tuberculosis: a pictorial review. Heliyon. (2024) 10:e29779. doi: 10.1016/j.heliyon.2024.e29779, 38699716 PMC11063446

[40] Zhang H Hasan T Dotel R Ulbricht E Gilroy N Maddocks S. Central nervous system tuberculosis in Western Sydney: a 10-year retrospective cohort study. Intern Med J. (2025) 55:822–32. doi: 10.1111/imj.70017, 40104936 PMC12077587

[41] Kumar I Shekhar S Yadav T Aggarwal P Singh PK Shukla RC . The many faces of intracranial tuberculosis: atypical presentations on MRI—a descriptive observational cohort study. Egypt J Radiol Nucl Med. (2023) 54:116. doi: 10.1186/s43055-023-01061-6

[42] Schweitzer F Laurent S Cortese I Fink GR Silling S Skripuletz T . Progressive multifocal leukoencephalopathy: pathogenesis, diagnostic tools, and potential biomarkers of response to therapy. Neurology. (2023) 101:700–13. doi: 10.1212/WNL.0000000000207622, 37487750 PMC10585672

[43] Al Malik YM. Tumefactive demyelinating lesions: a literature review of recent findings. Neurosciences. (2024) 29:153–60. doi: 10.17712/nsj.2024.3.20230111, 38981633 PMC11305340

[44] Cortese I Reich DS Nath A. Progressive multifocal leukoencephalopathy and the spectrum of JC virus-related disease. Nat Rev Neurol. (2021) 17:37–51. doi: 10.1038/s41582-020-00427-y 33219338 PMC7678594

[45] Baldassari LE Wattjes MP Cortese ICM Gass A Metz I Yousry T . The neuroradiology of progressive multifocal leukoencephalopathy: a clinical trial perspective. Brain. (2022) 145:426–40. doi: 10.1093/brain/awab419, 34791056 PMC9630710

[46] Ingebrigtsen SG Myrmel KS Henriksen S Wikran GC Herder M Tylden GD . Transient biopsy-proven progressive multifocal leukoencephalopathy-immune reconstitution inflammatory syndrome (PML-IRIS) in an elderly woman without known immunodeficiency: a case report. BMC Neurol. (2024) 24:436. doi: 10.1186/s12883-024-03945-0, 39521972 PMC11549778

[47] Piñar Morales R Carrasco Garcia M Gutierrez-Rojas L Barrero Hernández FJ. Progressive multifocal leukoencephalopathy and immune reconstitution inflammatory syndrome after discontinuation of fingolimod. Case Rep Neurol. (2022) 14:38–43. doi: 10.1159/000521944, 35350291 PMC8921886

[48] Grinspoon SK Fitch KV Zanni MV Fichtenbaum CJ Umbleja T Aberg JA . Pitavastatin to prevent cardiovascular disease in HIV infection. N Engl J Med. (2023) 389:687–99. doi: 10.1056/NEJMoa2304146, 37486775 PMC10564556

[49] Murala S Nagarajan E Bollu PC. Infectious causes of stroke. J Stroke Cerebrovasc Dis. (2022) 31:106274. doi: 10.1016/j.jstrokecerebrovasdis.2021.106274 35093633

[50] Schaefer JH Stephan C Foerch C Pfeilschifter W. Ischemic stroke in human immunodeficiency virus-positive patients: an increasingly age-related comorbidity? Eur Stroke J. (2020) 5:252–61. doi: 10.1177/2396987320927672, 33072879 PMC7538766

[51] Lalla R Raghavan P Cole JW. Extracranial ectasia and embolic infarcts in HIV: two case reports and a clinical decision-making algorithm. J Neurovirol. (2020) 26:474–81. doi: 10.1007/s13365-020-00867-8 32632673 PMC7470187

[52] Oliveira R Mendonça M Teodoro T Morgado C. Revisiting magnetic resonance imaging gadolinium contrast enhancement in subacute cerebral infarction: two cases leading to misdiagnosis. Prim Care Companion CNS Disord. (2023) 25:22cr03422. doi: 10.4088/PCC.22cr03422 37347672

[53] Beylerli О Talybov R Musaev E Trofimova T Shi H Ilyasova T . Cerebrovascular disorders in patients with malignant tumors. Brain Hemorrhages. (2024) 5:284–92. doi: 10.1016/j.hest.2024.08.003

[54] Syed W Ibatullin M. Glioblastoma: overview and magnetic resonance spectroscopy analysis for treatment. Cureus. (2024) 16:e66390. doi: 10.7759/cureus.66390, 39247004 PMC11379102

[55] Talybov R Beylerli O Mochalov V Prokopenko A Ilyasova T Trofimova T . Multiparametric MR imaging features of primary CNS lymphomas. Front Surg. (2022) 9:887249. doi: 10.3389/fsurg.2022.887249, 35510125 PMC9058099

[56] Talybov R Trofimova T Mochalov V Karasev S Gorshkova V Kleschevnikova T . The determination of the boundaries and prediction the radicality of glioblastoma resection using MRI and CT perfusion. Front Neurol. (2025) 16:6682–98. doi: 10.3389/fneur.2025.1572845, 40438576 PMC12116550

[57] Lin X IRA K YHC S Lee HY Yu WY. Atypical radiological findings of primary central nervous system lymphoma. Neuroradiology. (2020) 62:669–76. doi: 10.1007/s00234-020-02377-0 32077984

[58] Yu X Hong W Ye M Lai M Shi C Li L . Atypical primary central nervous system lymphoma and glioblastoma: multiparametric differentiation based on non-enhancing volume, apparent diffusion coefficient, and arterial spin labeling. Eur Radiol. (2023) 33:5357–67. doi: 10.1007/s00330-023-09681-2, 37171492 PMC10326108

[59] Abdalkader M Xie J Cervantes-Arslanian A Takahashi C Mian AZ. Imaging of intracranial infections. Semin Neurol. (2019) 39:322–33. doi: 10.1055/s-0039-1693161 31378868

[60] Karasev S Talybov R Chertoyev S Trofimova T Mochalov V Kleshchevnikova T . Diagnostic challenges of gliosarcoma: case report of a rare glioblastoma histopathological variant. Front Radiol. (2025) 5:1687401. doi: 10.3389/fradi.2025.1687401 41158141 PMC12554621

[61] Zou L Diao Y You C. Massive intracranial lesion in an AIDS patient: diagnostic challenge between brain tumor and toxoplasmic encephalitis resolved by empirical therapy. Int Med Case Rep J. (2025) 18:1527–32. doi: 10.2147/IMCRJ.S553371, 41362698 PMC12682301

[62] Batalov AI Zakharova NE Pronin IN Belyaev AY Pogosbekyan EL Goryaynov SA . 3D pCASL-perfusion in preoperative assessment of brain gliomas in large cohort of patients. Sci Rep. (2022) 12:2121. doi: 10.1038/s41598-022-05992-4, 35136119 PMC8826414

[63] Feng A Li L Huang T Li S He N Huang L . Differentiating glioblastoma from primary central nervous system lymphoma of atypical manifestation using multiparametric magnetic resonance imaging: a comparative study. Heliyon. (2023) 9:e15150. doi: 10.1016/j.heliyon.2023.e15150, 37095995 PMC10121909

[64] Kizaki T Kanazawa M Ishiguro T Natsumeda M Tada M Shimizu H . Indications for a brain biopsy in neurological diseases of unknown etiology: the role of magnetic resonance imaging findings and liquid biopsy in yielding definitive pathological diagnoses. J Neurol Sci. (2024) 463:123150. doi: 10.1016/j.jns.2024.123150, 39067261

[65] Ismael S Moshahid Khan M Kumar P Kodidela S Mirzahosseini G Kumar S . HIV associated risk factors for ischemic stroke and future perspectives. Int J Mol Sci. (2020) 21:5306. doi: 10.3390/ijms21155306, 32722629 PMC7432359

